# Transformation starts at the periphery of networks where pushback is less

**DOI:** 10.1038/s41598-024-61057-8

**Published:** 2024-05-18

**Authors:** Ingrid A. van de Leemput, Jordi Bascompte, Willem Bastiaan Buddendorf, Vasilis Dakos, J. Jelle Lever, Marten Scheffer, Egbert H. van Nes

**Affiliations:** 1 Department of Environmental Sciences, Wageningen University and Research, Wageningen, The Netherlands; 2 Department of Evolutionary Biology and Environmental Studies, University of Zurich, Zurich, Switzerland; 3 Wageningen Environmental Research, Wageningen, The Netherlands; 4 Institute Des Sciences de L’Évolution, Université de Montpellier, CNRS, IRD, EPHE, Montpellier, France; 5 Swiss Federal Institute for Forest, Snow and Landscape Research (WSL), Birmensdorf, Switzerland

**Keywords:** Network, Tipping elements, Cascades, Systemic shift, Resilience, Ecological networks, Ecology, Psychology, Systems biology, Dynamical systems

## Abstract

Complex systems ranging from societies to ecological communities and power grids may be viewed as networks of connected elements. Such systems can go through critical transitions driven by an avalanche of contagious change. Here we ask, where in a complex network such a systemic shift is most likely to start. Intuitively, a central node seems the most likely source of such change. Indeed, topological studies suggest that central nodes can be the Achilles heel for attacks. We argue that the opposite is true for the class of networks in which all nodes tend to follow the state of their neighbors, a category we call two-way pull networks. In this case, a well-connected central node is an unlikely starting point of a systemic shift due to the buffering effect of connected neighbors. As a result, change is most likely to cascade through the network if it spreads first among relatively poorly connected nodes in the periphery. The probability of such initial spread is highest when the perturbation starts from intermediately connected nodes at the periphery, or more specifically, nodes with intermediate degree and relatively low closeness centrality. Our finding is consistent with empirical observations on social innovation, and may be relevant to topics as different as the sources of originality of art, collapse of financial and ecological networks and the onset of psychiatric disorders.

## Introduction

Much network theory focuses on the effects of topology, i.e., the specific ways in which elements (nodes and links) are arranged. This approach allows inferring interesting properties purely from the structure of connections. For instance, it can be shown that in scale-free communication networks, where a few nodes have disproportionally many connections (i.e., hubs), removal of a random node does little damage to the overall connectivity of the network, as there are many alternative pathways from one node to another. By contrast, taking out one of the central nodes has a disproportionately large damaging effect. Thus, the functioning of such networks is undermined only when a highly connected central node is attacked^1^. Others (e.g^2^) have focused on the effects of node or edge removal on the overall resilience of a network.

In reality, however, networks are often affected by perturbations that are more subtle than the removal of entire nodes or links. In many networks, changes in the state of a single node may lead to cascades of change on other nodes. In social networks, for instance, people tend to adopt a similar attitude as their peers on a topic^3,4^ or in tropical forests, moisture recycling by forested patches may prevent cascading collapse^5,6^. Comparable mechanisms operate in financial networks, where failure (collapse due to defect or bankruptcy) of a bank can draw connected banks into failure^7,8^, whereas for banks embedded in a majority of healthy banks, cascading failure becomes less likely. Similarly, in metapopulations local extinction risks are strongly reduced due to immigration from neighboring sites^9,10^, a phenomenon called the ‘rescue effect’. Such rescue effects reduce the chance of collapse of a single sub-population, but may also pave the way for a large-scale systemic shift in the state of the entire network when a tipping point is passed^9^. Another, seemingly unrelated, example is the network of symptoms in psychiatric disorders. For instance, depression causes subjects to ruminate, sleep badly, take less physical exercise, retreat socially, and eat worse. These symptoms are causes too as they promote each other thus deepening the overall depressed state^11,12^. Such vicious circles may be broken by cognitive-behavioral therapy directed at certain elements (e.g., going for a long walk daily) which can then "pull" other elements back towards the healthy state.

We may call the type of network in those examples ‘*two-way pull networks’*, because nodes in these networks have a mutual tendency to assume the same state as their neighbors. An implication on which we will elaborate later, is that well-connected nodes of the core are protected from shifting by the pull-back effect from their neighbors. Not all networks have such a two-way pull character between nodes. For instance, the spread of epidemics has a one-way pull force only. As Aristotle famously noted in his collection of puzzling observations (the ‘Problemata’), a healthy person can become ill through contact with sick persons. However, bringing a sick person in touch with healthy ones does not make a sick person healthy. Obviously, a central position in a social network does not help to prevent a well-connected person from falling ill. Instead, they may function as potential ‘super-spreaders’ once they are sick^13^. We may see such one-way pull effects as the extreme end of a spectrum. From these preliminary observations we may infer that the degree of asymmetry in pull forces will influence whether the core or the periphery are the most likely starting places for change. For instance, a brilliant innovation that is obviously useful will probably spread wherever it starts in a network. By contrast, controversial or marginally beneficial innovations may or may not make it depending on where they originate in a network as transformed persons may be pulled back to the majority view^14^.

Here we systematically analyze the question of where cascading change is most likely to start in two-way pull networks. We analyze models of networks with different structures demonstrating that under a broad range of conditions the center is relatively inert, while changes in moderately connected nodes in the fringe are the most likely origin of transformation. We discuss how this corresponds to empirical findings from different fields, and formulate applied research questions for each of those fields.

## Approach

To study from which part of a two-way pull network, a systemic transition (that affects all nodes in the network) is most likely to start, we used simulation experiments perturbing each of the nodes in a network one by one and monitoring the effect on the network as a whole. We did this for a large collection of networks with the same number of nodes but different degree distributions of the nodes (architectures). We created this collection using computer algorithms generating four different sets of networks: random networks, exponential networks, scale-free networks and random-regular networks (details in online appendix S2). Random networks have a binomial degree distribution, while exponential and scale-free networks have much more right-skewed distributions of degrees (respectively exponential and power law distributions). In random-regular networks the degree of each node is forced to be fixed.

The dynamics of the nodes of these networks were modeled using two contrasting models: the deterministic Allee effect model^15^ and the stochastic Ising model^16^. Hereafter we refer to those in short as the deterministic and stochastic model. For the deterministic model, we defined the dynamics of the state of each of the nodes *i* in a network as a continuous variable (*X*_*i*_) affected by the state of connected nodes (*X*_*j*_):

1 $$\frac{d{X}_{i}}{dt}={r}_{i} {X}_{i}\left(1-\frac{{X}_{i}}{{K}_{i}}\right)\left(\frac{{X}_{i}}{{C}_{i}}-1\right)+\left(\sum_{j}{I}_{ij}{X}_{j}-\left(\sum_{j}{I}_{ij}\right){X}_{i}\right) d-{m}_{i} {X}_{i}$$

This is mathematically equivalent to classical models used to describe the dynamics of subpopulations *X*_i_ (with growth rate (*r*_*i*_) and carrying capacity (*K*_*i*_) both set to unity for all subpopulations) connected in a network where they form a so-called metapopulation. Each subpopulation in a node has an Allee effect^15^ of which the strength is defined by *C*_*i*_*.* This effect accounts for the phenomenon that a subpopulation in isolation cannot survive when it becomes too small. As a result, each individual node is a ‘tipping element’ that in isolation may exhibit a ‘low-biomass’ and a ‘high-biomass’ alternative stable state. In a network the stability and biomass in both of these states also depends on interactions with neighboring nodes. The structure of the network of interactions is given by a symmetric adjacency matrix describing the interactions of the network (*I*_*ij*_ = *I*_*ji*_ and* I*_*ii*_ = *0*, where 1 = connection and 0 = no connection). The interactions have the nature of a diffusive exchange depending on the difference in state (*X*_*j*_*—X*_*i*_) between two nodes scaled by the coupling strength (*d*). This two-way exchange results in a tendency of the state of any node to move towards the state of neighbors. Lastly, there is a loss parameter (*m*_*i*_) that corresponds to a stress on the system. This parameter reduces the overall resilience of the high-biomass state increasing the likelihood of collapse into the low-biomass state. Except for the interaction terms (*I*_*ij*_), all parameters were the same for each of the nodes (Table S1). However, in the Supplementary Information we explore effects of heterogeneity by assigning different carrying capacities (*K*_*i*_) to individual nodes drawn from a uniform distribution.

At the start of the perturbation experiment, we set all nodes to the ‘high-biomass state’. We then perturb each of the nodes one-by-one and analyze for each node how vulnerable the network is to perturbation of this node. We do that by finding the critical value of parameter *m* that allows a perturbation of that node to cascade through the entire network. We thus find the node-specific critical value of this loss-parameter for a systemic collapse. The perturbations consisted of setting the biomass of the target node to the low-biomass state. In addition, we studied effects of a local ‘press perturbation’ where (in addition to lowering biomass) we locally fix the loss parameter (*m*_i_) of the perturbed node to a high value preventing the target node from recovering.

We proceed by analyzing how the vulnerability of the network to a particular perturbed node depends on the connectivity of that node in the network. We study two aspects of this connectivity: (1) *degree*, defined as the number of direct links of a node with other nodes, and (2) *closeness centrality* reflecting the distance, in terms of number of links, between a single node and all other nodes . The closeness centrality of node *j (CC*_*j*_*)*^17^ is defined as the reciprocal of the mean shortest path between this node and all other nodes:

2 $${CC}_{j}=\frac{n-1}{\sum_{j,j\ne i} {D}_{i,j}}$$

where *n* is the number of nodes of the network and *D*_*i,j*_ is the shortest path (in number of links) between nodes *i* and *j*, where $i\ne j$.

To probe the generality of our results we also analyzed a stochastic model, the ‘Ising model’, which was originally developed to describe the polarization of a ferromagnet^16^, but has also been applied to study mechanisms behind shifts in public opinion and many other problems^18^. In this stochastic model the nodes (i.e. the magnetic dipoles) have a probability to shift between two distinct alternative states (a ‘spin’ of either + 1 and -1) depending on the spins of neighboring nodes, an external magnetic field and the temperature (for details see Supplementary Information S1). The model was tuned such that the system occasionally collapsed, such that single-node perturbation experiments would be able to trigger a large-scale change. Similarly to the deterministic model, we experimented by perturbing each of the nodes in two different ways: flipping the state of the studied node only, or flipping the state combined with a press perturbation by increasing the external magnetic field locally (parameter *h*_*i*_). As this model is stochastic, one cannot determine the exact critical level of fragility of the network as in the other deterministic model. Instead, we used large sets of simulation experiments to estimate the probability of shifting more than half of the nodes as a measure of resilience (see Supplementary Information S1).

## Results

**Figure 1:**
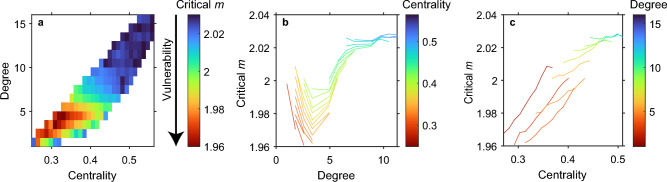
Effect of centrality and the number of connections (‘degree’) on the vulnerability of a node to trigger a systemic transition in the network, based on exponential networks of the deterministic Allee effect model. (**a**) Combined effects of centrality and degree. The color represents the critical value of parameter *m* at which a perturbation of a node causes a systemic transition, based on 2D interpolation. Note that a low critical value of the loss parameter *m* implies a high vulnerability (indicated by the arrow). The red hotspot indicates the combination of degree and centrality of nodes from where change starts most easily. (**b**) The effect of the degree of the perturbed node. Each curve represents the results for a given bin of centralities. (**c**) The effect of centrality of the perturbed node, with curves representing nodes within a given bin of degrees. Parameter *d* = 0.15 (other parameters as in Table S1). Average critical *m* values in plots (**b**) and (**c**) are plotted only if the value is based on at least 10 nodes.

Our analysis of the deterministic Allee effect model reveals that a systemic shift is more likely to arise from perturbation of nodes in the periphery of a network, especially if those nodes have an intermediate level of connectivity. Such an optimum level of connectivity is found if we consider the number of direct links (i.e. degree of the node), but also if we look at the number of indirect links as reflected in the closeness centrality (Fig. 1, and Figures S1–S6).

Before getting to the mechanisms explaining those dynamics, it is worth noticing that those qualitative patterns are rather robust across parameter values and network architectures. For instance, when the coupling between nodes is weaker (smaller *d*), the effects remain although being less prominent (Figures S1 -S3). Similarly, when the initial node is pushed to a persistent alternative state rather than flipped in a single event (see methods), the qualitative effects of centrality and number of neighbors remain unaltered (Figures S4-S6). Also, for different network architectures (random networks, exponential networks, and scale-free networks) the results are qualitatively similar (Figures S1-S6). Lastly, the results also hold for mildly heterogeneous networks, if we allow the parameter *K* (affecting resilience of nodes) to vary by 10%. Not surprisingly, if differences between the *K* values of nodes become too high, the occurrence of a systemic shift becomes independent of the location of the initial perturbation (Figure S7).

Obviously, centrality and connectivity are not independent as more central nodes will often have a higher number of direct neighbors (degree) too. We therefore explored their effects independently by visualizing the effect of degree within each class (bin) of centrality (Fig. 1b, Figures S2 and S5) and the effect of centrality within each class of degree (Fig. 1c, Figures S3 and S6). The existence of a sweet spot of connectivity for triggering systemic change is even more clear from these figures. To double-check whether the effect of centrality is really independent from the effect of the number of neighbors, we generated a set of random-regular networks, a special kind of random network where each node has the same number of connections, but can have a different centrality. Perturbations of these random-regular networks confirm that independently from any degree-effect, change spreads more easily from nodes in the periphery than the center of a network (see Figure S11).

**Figure 2:**
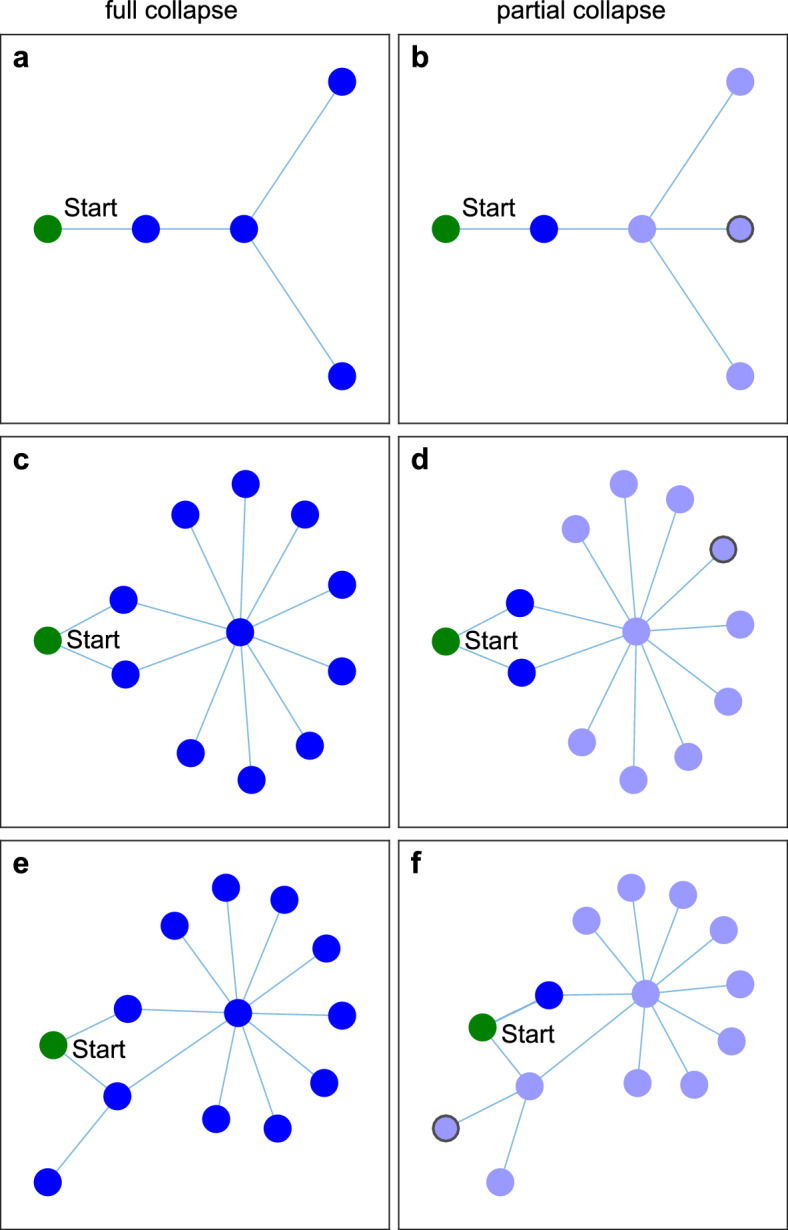
Local barriers to cascading spread of a perturbation in small constructed networks (deterministic Allee effect model). At the start of the simulation all nodes are in the high biomass equilibrium except for the green node indicated with ‘Start’, which has no biomass. Left (panels a,c,e) are networks where all nodes collapse, right (panels b,d,f) are the same networks but with one extra node (indicated with a grey outline) that causes the cascading spread to halt. The light blue nodes stay in the high biomass state; the dark blue nodes are collapsed together with the start node (green). When the perturbation initially spreads to a few low connected nodes, they can act together to let a common highly connected neighbor collapse. Parameter values: *m* = 1.99; *d* = 0.05 (other parameters as in Table S1).

To understand the underlying mechanism behind this unimodal effect of centrality and degree we perturbed some very simple networks in search for bottlenecks that may prevent the spread of a perturbation (Figs. 2 and Figure S12). Those analyses suggest that at the roots of the phenomena we found is a kind of critical mass balance for the local spread of change. In Fig. 2 we show how adding one node to a network can help prevent a systemic shift. For instance, a perturbation in a single node with one neighbor can only spread if that neighbor is connected to at most two other nodes (Fig. 2a,b). However, if two shifted nodes can cooperate, together they may turn over a common neighbor that has at most 8 other neighbors (Fig. 2c,d) and is thus much more connected. Figure 2e,f show that the two cooperating nodes can have maximal 1 additional neighboring node themselves to be shifted by the initially perturbed node. By contrast, if the initially perturbed node is highly connected, its state shift cannot spread (Figure S12b) and is often even directly reversed by the common effect of the neighbors (Figure S12c). Those observations help to understand why the likelihood of a systemic shift is largest if a perturbation starts in a moderately connected node in the fringe, and suggest that this is especially so if this initial node is connected to other weakly connected nodes. Such situations may prevent stagnation of the propagation of change at barriers where the critical mass balance between the two states cannot be overcome.

**Figure 3:**
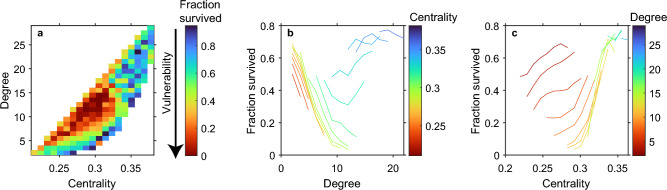
Results as in Fig. 1, but now based on a stochastic model describing the orientation of magnetic spins in a network (an Ising model, exponential network, see Supplementary Information S1). Here, a press perturbation is performed, with the external magnetic field (*h*) of the perturbed node set to 10. The chance that a transition starts in a node of a given centrality and degree is expressed as the average fraction of simulations in which the network remained in the same state despite the perturbation. (For methods see main text and Supplementary Information S1). Parameter values: *h* = 1.6 (other parameters as in Table S1). The average fraction survived in plots b and c is plotted only if the value is based on at least 10 nodes.

To further probe the generality of our results, we redid the analyses of the effect of centrality and degree using a very different kind of model, the stochastic Ising model originally developed to describe the behavior of magnetic particles^16^ (see Supplementary Information S1). All results from this model, often used to describe dynamics of opinion in social networks, were in line with those based on the deterministic continuous-state model (Fig. 3, Figures S8-S10), supporting the view that the patterns we find are robust against the choice of model formulation. In the Ising model, the patterns tend to be more noisy due to the stochastic runs of the experiments. Also, in the Ising model, perturbations of the state by changing the spin of a single node only are often not strong enough to cause a systemic shift (Figure S8), because an individual flip tends to be reversed immediately. This effect can be ameliorated by increasing the resilience of the perturbed node that creates a local press perturbation (Figure S8). In that case, we obtain results similar to those obtained with the other model formulation. Again, nodes in the periphery are the most likely starting points for a systemic shift. Interestingly, while in the deterministic model the parameter range in which a single node can trigger a shift is relatively small (i.e. the system has to be close to a tipping point), in the stochastic model the results are more generic.

In conclusion, our results suggest that transformation in two-way pull networks is more likely if one causes a systemic shift in the periphery, than if one targets a central node.

## Relation to other theoretical results

We scanned the literature to see how our results relate to those from other theoretical studies, but surprisingly, the question of where cascading change is most likely to start in push–pull networks is rarely addressed dynamically.

There are extensive literatures on spread of attitudes in social networks. For instance, a substantial body of research has been inspired by the strategic question of which individuals should be recruited to influence the largest audience in marketing contexts. For this purpose social influence maximization models are applied to various synthetic or observed network structures, using different assumptions on the distributions of susceptibility and influential power of individuals^19^. Also, indicators of centrality have been proposed that take ‘complex contagion’ into account^20,21^. The idea in all such approaches is that there is some form of peer reinforcement, an individual is more likely to be convinced (e.g. of buying a product or signing up to a forum) if this is suggested by more ‘friends’. In line with our results, Guilbeault & Centola^21^ conclude that social innovations are more likely to spread from the periphery than from the center of a network, if complex contagion is taken into account. However, none of those approaches takes the symmetric push–pull nature of contrasting states into account that we addressed in our study. Rather, they look at a unidirectional spread that may be stopped, but not reversed. As argued this can be an essential characteristic of networks of entities such as psychological symptoms, financial institutions, or coral reefs, but also for social networks when it comes to competing attitudes towards contested issues (such as gender fluidity or climate change).

A notable exception is an early simulation study by David Krackhardt on the spread of ‘controversial innovations’ in organizations^14^. Krackhardt lets individuals in his model search locally in the organization network to see if anyone agrees with a suggested innovation. Only if they find no one agreeing they ‘give up’ and change their opinion and stop supporting the suggested innovation. Krackhardt finds that a change will be most likely adopted throughout the organization if the early adopters form a cluster at the periphery. He also finds an optimal level of network coupling strength (which he calls ‘viscosity’) in terms of how easily people would move to explore opinions in other clusters within the organization. If this coupling strength is too low the innovation remains isolated. If it is too high, then the larger group of nonadopters will flood the innovative cluster causing return to the status quo. Only an intermediate level of viscosity allowed the early adopters to convert nonadopters at a greater rate than the converse, eventually causing the entire organization to adopt the innovation. Clearly, those results are well in line with our general findings and illustrate how naturally this way of thinking may be applied to the issue of social innovation as discussed in some more detail in the next section.

Although we could find no studies specifically asking where change starts most easily in two-way pull networks, we did find two studies that showed the effect of the number of connections (the degree) of a disturbed node. One study used an information-theoretical approach, to show that for random networks the impact on the short-term behavior of the whole system is lower if the perturbation is applied to a high degree node, while Ising network simulations suggested a unimodal effect of connectivity on the time it took for the effect of perturbation of a node to fade away^22^. Other authors found that in networks of oscillators ‘failure’ of low-degree nodes has most impact on networks in which the active units could compensate for the failure of neighboring (inactive) units at the expense of a reduction in their own activity^23^. Both studies thus support the idea that network change is generally easier to start from moderately connected nodes. However, note that the effect of centrality we find comes on top of such an effect of degree, as within a group of nodes with the same degree, less central nodes have on average a stronger effect (Figure S10).

In conclusion, while the question of where transitions start most easily in push–pull networks has received little attention, the few studies that are available generally point in the same direction, supporting the view that the key predictions are robust against the particular kind of model used.

## Empirical evidence

It is challenging to match the clean results from theoretical models to patterns found in real world situations, which are inevitably much more complex and heterogeneous. Nonetheless, observations from a diverse set of systems seem to support the idea that change may start more easily in the periphery rather than at the center.

### Sources of transformative innovation in social networks

Classical work on innovation predominantly emphasizes the role of the cities and headquarters of transnational corporations as the places where new things emerge^24^. However, there is a rising interest in the role of the periphery as generator of novelty^25^. The idea that innovation may start in the periphery where it is shielded from flooding by the dominant points of view is indeed intuitive. But is there empirical support for it? Quantitative research is difficult in this field, but various qualitative studies seem to support the idea that novelty may arise in the periphery. One example comes from a case study of innovation in the multinational BASF company^26^. Systematic interviews and a social network survey of knowledge-sharing among employees revealed how an important controversial innovation came from the company’s Argentinian node thanks to its peripheral position. The loose and leaky connectivity to the core was essential as it provided opacity caused by some degree of miscommunication which allowed the controversial idea to persist and develop in the periphery^26^.

Another study looked into the factors explaining the success of Broadway musicals. It turned out that the most successful musicals were written by teams that had neither too much nor too little connectivity to the larger creative network, as measured through a ‘small-world index’^27^. The idea here is that links help the spread of fresh creative material, but too much connectivity gives everyone the same pool of creative material. Also, too many ties decrease the artists’ ability to break away from conventional ideas or styles that may have worked in the past but now lost their appeal.

The Broadway example is reminiscent of the deliberate strategy of the famously innovative Nobel laureate Richard Feynman who sought a certain level of disconnect from existing ideas by avoiding reading the literature too well and seeking ways of circumventing the usual approaches^28^. While this is rather anecdotal evidence, the nature of scientific publishing with its massive databases of publications and reference structures now also allows quantitative studies hinting at potentially inhibitory effects of connectivity when it comes to innovation. For instance, automated analysis of over 65 million papers, patents, and software products^29^ revealed that smaller teams have tended to disrupt science and technology with new ideas, whereas larger teams mostly developed existing ideas further. While causality is hard to infer, the authors suggest that it could be related to the fact that in larger groups individuals tend to generate less ideas, reject external perspectives more often and neutralize each other’s viewpoints.

None of those examples allows separating out the effects of centrality specifically, as we did with the systematic analyses of models. However, all examples do support the idea that novelty can be stifled if its source is embedded too strongly in a web dominated by the status quo.

## Outlook

Patterns of social innovation support our theoretical prediction that change may tend to start from the periphery rather than in the core of networks. This implies that the prevailing view that central nodes are key to triggering network change may need to be revised in two-way pull networks. This view is largely based on topological analyses that study the topological effect of removing nodes from a network altogether (e.g. destroying a specific powerplant in a grid). Also, the ‘vulnerability at the core’ makes sense in case of unidirectional effects such as the propagation of a disease in an immunologically naive population^30^, or success of marketing through influencers^31^. However, clearly there are many situations in which change is the more subtle outcome of two-way pull forces between neighboring nodes. The empirical cases we discussed are just a subset where some evidence was available, but two-way pull forces may govern change in many other networks, including the cascading failure among financial institutions^7,8^, networks of mutually reinforcing psychiatric symptoms^11,12^, cascading failure of organs of critically ill patients^32,33^ or the collapse and recovery in networks of local ecosystems such as the thousands of individual reefs in the Australian Great Barrier Reef system ^34,35^. Our results provide a search image in the quest to spot the Achilles heel of complex networks. At the same time they may put us on the track of the most promising starting points for positive change^36^ such as cascading shifts in social norms that may help shifting the globe to more sustainable pathways^37^, or cascading effects of local restoration of degraded ecosystems^38^. Clearly, our models remain highly abstract, and understanding any particular case will require follow-up work tailored to its specific characteristics. To test our hypothesis in more realistic settings, it would be useful to perform similar tests in real world network models, or in experiments. As a starting point we list some open questions (Table 1) that might inspire such a quest in different systems.

**Table 1:** Systems where we can expect vulnerability near the edge. Specific questions for follow-up research.

System	background	question
Psychiatric disorders	Many psychiatric disorders correspond to networks of symptoms such as insomnia, rumination, and depressed mood that are mutually reinforcing^11,12^	Are central symptoms in such networks more resistant to treatment?
Meta-populations	Meta-populations or ecosystems such as coral reefs or remains of forests often occur in a spatial network of patches where migration between the patches enhances resilience and creates a ‘rescue effect’^9,10,35,39,40^	Should protective measures, but also restoration efforts, focus on the peripheral rather than the core patches?
Science	It may be more difficult to get an entirely different idea if you build on the same information^41^	Is there an optimal level of disconnect between research groups?
Art	Artists are influenced by their peers and surroundings^27,41^	Is art from isolated artists more original?
Power grids	From a single element, failure may cascade through power grids as loads are shifted to nearby elements which are then pushed beyond capacity^42,43^	Is failure due to overload more likely to start from a peripheral element?
Financial networks	Banks and other financial institutions depend on each other through a partly invisible network of loans and other agreements^8,44^	Is systemic failure most likely to start from peripheral banks?

### Supplementary Information

Supplementary Information.

## Data Availability

Code for simulations have been deposited on Gitlab: https://git.wur.nl/sparcs/network_tipping.
