# Supplementary material for: Transformation starts at the periphery of networks where pushback is less
Source: Sci Rep. 2024 May 18;14:11344. doi: 10.1038/s41598-024-61057-8 (PMC11102466; doi:10.1038/s41598-024-61057-8)
Supplement: Supplementary file 1 — Supplementary Information. [file 41598_2024_61057_MOESM1_ESM.pdf]

Supplementary information

Transformation starts at the periphery of networks where pushback is less

Ingrid A. van de Leemput, Jordi Bascompte, Willem Bastiaan Buddendorf, Vasilis Dakos , J. Jelle Lever, Marten Scheffer, and Egbert H. van Nes

S1 The stochastic Ising model

The classical Ising model [46] originally described the polarization of a ferromagnet, but is also used in various modelling studies representing other types of networks, for instance psychologic disorders [47], scientific theory [48], and public opinion about environmental problems [49]. Here we use a generalized network version, where each node of the network is a discrete unit that can have 2 spin states: up (+1) or down (-1). Each of these configurations has a certain energy that depends on the state of the neighbors. The system tends towards a lower energy configuration, but there are also stochastic perturbations. The energy of a certain configuration is defined by the Hamiltonian *H(σ)* (minus signs are a convention).

$$H(\sigma) = -\sum_{<i,j>} J_{i,j}\, S_i\, S_j \,-\, \sum_j h_j S_j \qquad \text{(S1)}$$

where $J_{i,j}$ is the interaction strength between 2 neighboring cells (default J=1)

$<i,j>$ = all pairs of nearest neighbors in the network

h_j = external magnetic field at node j

S_i = the spin of cell i in the lattice.

The law of Boltzmann (for instance also valid for gasses) couples the energy of a certain configuration *H(σ)* to the probability that it exists:

$$P(\sigma) = \frac{e^{-\beta\, H(\sigma)}}{\sum_j e^{-\beta\, H(\sigma_j)}} = \frac{e^{-\beta\, H(\sigma)}}{Z} \qquad \text{(S2)}$$

where β = 1/(k*T) where T is temperature in Kelvin, k is the Boltzmann constant (we arbitrarily choose β=1), and

$Z = \sum_j e^{-\beta\, H(\sigma_j)}$ is called the partitioning function, representing the sum of powers of all possible configurations.

Although this Z factor is very tedious to determine as we need to sum all 2^N configurations, this equation is the basis of all analyses of the Ising model. We use the Metropolis algorithm [50] to update the cells one-by-one in random order, testing whether a flipped state has a lower energy. For each node we calculate the change in energy for flipping the spin using equation S3

$$\Delta H_i = 2\, S_i \sum_j J_{i,j}\;\, S_j + 2\, h_i\, S_i \qquad \text{(S3)}$$

S_i is the spin of node i. $J_{i,j}$ is the interaction strength between node i and j ($J_{i,j}$=1 for a connection and $J_{i,j}$=0 if there is no connection).

If the change in energy is negative ($\Delta H \leq 0$) the node will always flip, otherwise there is a probability that the node will flip to a higher energy state (probability $e^{-\beta\,\Delta H}$). Note that this implies that if the number of neighbors > $|h|$, one node with opposite spin to the external field will always flip back when it is updated.

For efficiency, we simultaneously update a random fraction of 10% of the nodes in each time step, which is small enough to prevent erroneous cyclic behavior. We start simulations with all nodes in the less resilient state. We first tune the overall external magnetic field (h) for each kind of network to be close to the critical value, such that the system sometimes collapses in test runs. Then we draw a random network configuration of 500 nodes using the same parameters (see S2), and calculate the degree and closeness centrality of all nodes. Due to the stochastic nature of the model, we cannot use the same indicator of vulnerability based on full collapse as for the deterministic model. Therefore, we indicate the vulnerability of a perturbation of each node to trigger collapse by the fraction of runs in which <50% nodes have shifted after a simulation of 4000 time steps.

We perturb the studied node by reversing its spin. As this reversed spin is often quickly repaired, we keep the node continuously perturbed. For this, we set the "external magnetic field" h_i of the perturbed node i locally to a higher value (we used $h_i = 10$). After these perturbations we repeat a simulation of 4000 steps 100 times to estimate the average probability of survival. We repeat this for each of the other 500 nodes in the tested network and repeat this for 80 randomly drawn networks.

S2 The tested networks

We tested different network architectures that were generated at random. The following networks were generated:

Random network. We generate this network by drawing a uniformly distributed number for each element of the adjacency matrix and add a connection if this number is larger than a certain probability (parameter p) [51]. The edges $I_{i,j}$ are made bidirectional by copying $I_{i,j}$ to $I_{j,i}$.

Exponential network. In this network we use the algorithm of Albert and Barabási [52,53], start with one node and randomly add new nodes without preferential attachment. The average number of new nodes is defined by the parameter n_{legs}. This results in an exponential degree distribution.

Scale-free network. This network has a similar growing algorithm as the exponential network [51-53], but the probability of attachment scales with the number of links that already exist. The average number of new nodes is defined by the parameter n_{legs}. This results in a power-law degree distribution with few well connected hubs.

Random-regular network. A random network where all nodes have the same degree [54]. All nodes start empty and random pairs are selected from the nodes that have less connections than the target degree.

If a generated network had any isolated nodes, a new network was generated till all nodes were connected. For the deterministic Allee effect model we used 50 nodes and for the stochastic Ising model 500 nodes. The closeness centrality and degree of each node was calculated and all nodes were tested one-by-one. For the deterministic model, we analyzed 200 replicates for each kind of network. For the stochastic model we used 80 replicates, as the number of tested nodes per network was much larger.

Table S1. Default parameter values

Network architecture	symbol	Description	Default value
Random	p	Probability for adding a connection	0.1
Exponential	n_{legs}	Average number of new nodes	2.5
Scale-free	n_{legs}	Average number of new nodes	2.5
Random-regular		Degree of all nodes	4
**Model**	**symbol**	**Description**	**Default value**
Deterministic Allee effect model	X_i	State variable in node *i* (e.g. biomass of species)	
	K_i	High equilibrium in node *i* (e.g. carrying capacity of the species)	1
	r_i	Growth rate in node *i*	1
	C_i	The unstable equilibrium defining the Allee effect in node *i*	0.1
	d	Coupling strength between two nodes (e.g. exchange rate, diffusion)	0.05 or 0.5
	m_i	Loss parameter defining stability of node *i*	1.9-2
	I_{ij}	Adjacency matrix defining the network	0 or 1
Stochastic Ising model	S_i	The spin of cell *i* , can be +1 or -1	
	h_i	The external magnetic field of node *i*	[--]
	β	Inverse temperature	1
	$J_{i,j}$	Interaction strength between nodes *i* and *j*	1

S3 Supplementary videos

In these videos (https://git.wur.nl/sparcs/network_tipping) we show an example of a network and some examples of the perturbation of a single initial node in the deterministic Allee effect model. Kind of network: exponential network (n=50) with n_{legs}=2.5, *d*=0.05, *m*=1.992 (other parameters see Table S1). The lower panels show time plots of the closeness centrality and degree of the nodes that are collapsed.

Files:

- network_collapsed1.mp4 – example 1 of a successful systemic collapse, starting in node 7.
- network_collapsed2.mp4 – example 2 of a successful systemic collapse, starting in node 31.
- network_partial.mp4 – example of a partial collapse, starting in node 2.
- network_recovered.mp4 – example of an immediate repair, starting in node 50.

S4 Supplementary figures

Note we show for each scenario 3 figures (Figures S1, S2, and S3) with the same data

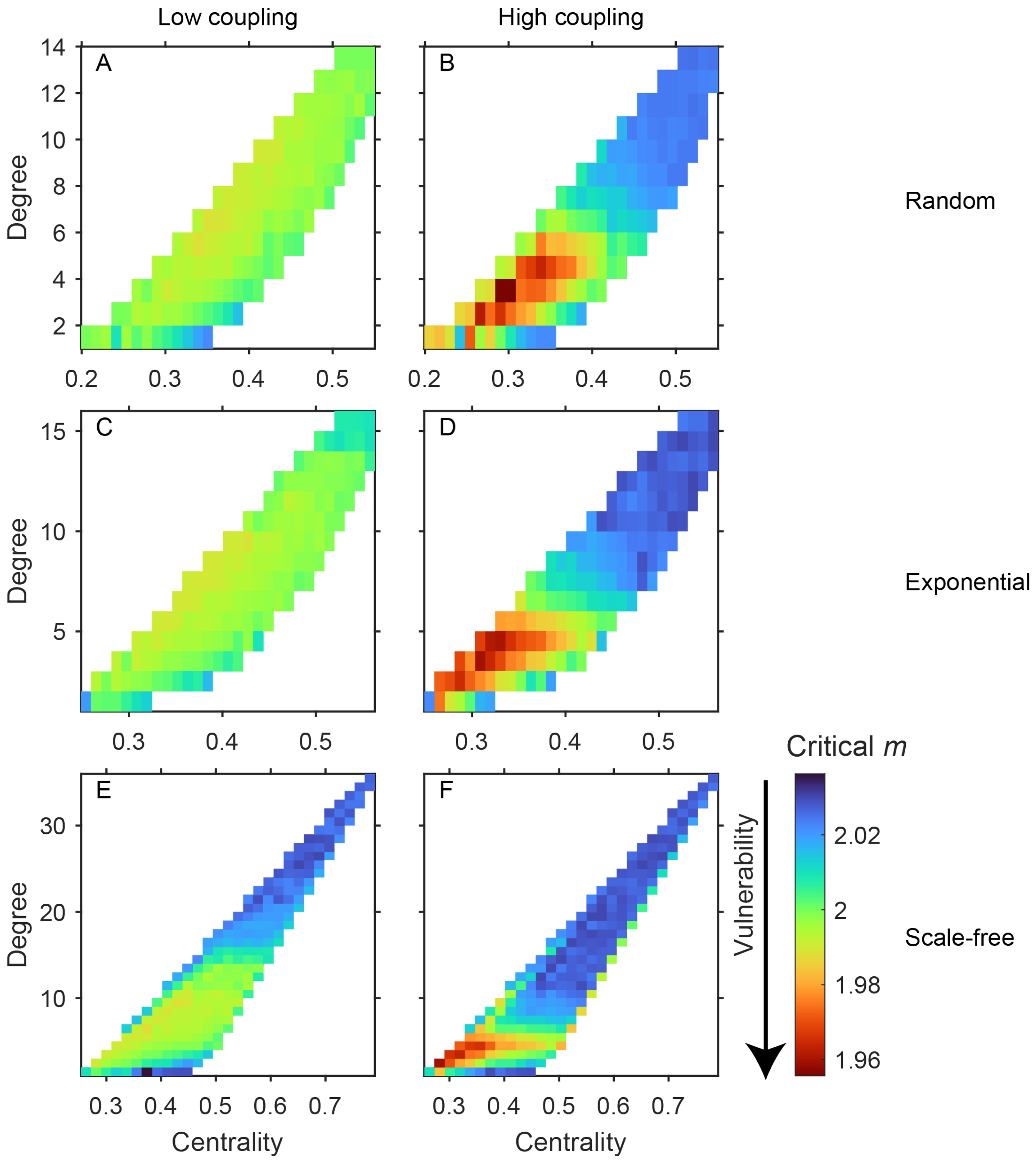

Figure S1. Effect of centrality and the number of connections ('degree') on the vulnerability of a node to trigger a systemic transition in the network. Different panels represent different network architectures of the deterministic Allee-effect model. The studied nodes are perturbed by a change of the state only. Red shading represents a high vulnerability in the sense that a systemic transition can be triggered from a node in this position even if the network is quite resilient (corresponding to a low value of parameter m_i in eq. 1). A. Random network with low coupling strength (d = 0.05); B. Random network with high coupling strength (d = 0.15). C. Exponential network with low coupling strength (d = 0.05); D. Exponential network with high coupling strength (d = 0.15). E. Scale-free network with low coupling

strength(d = 0.05); F. Scale-free network with high coupling strength (d = 0.15). Other parameter values as in Table S1.

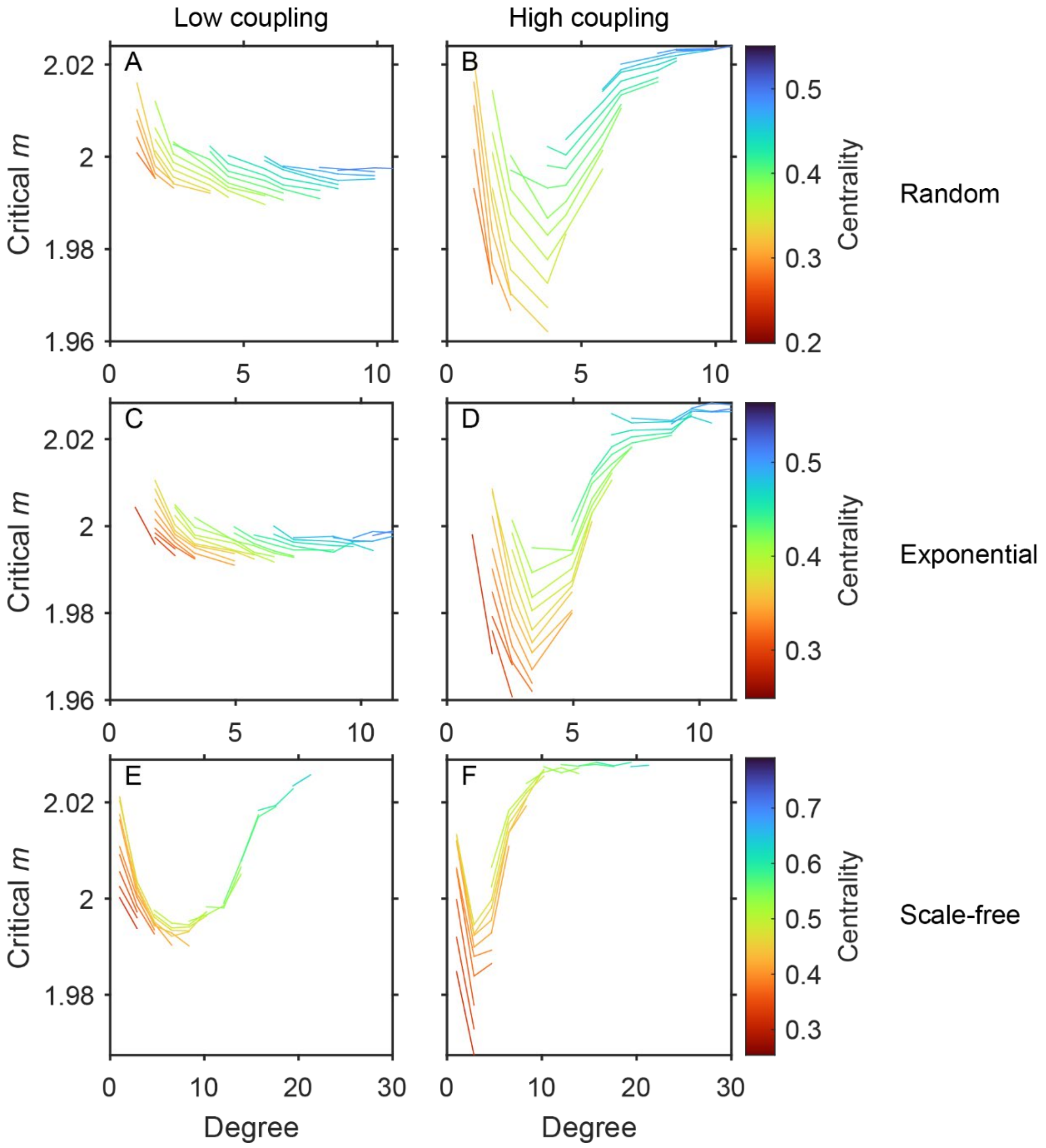

Figure S2. Results as in Figure S1, here represented as the effect of the degree of the perturbed node on the vulnerability. Each curve represents the results for a given bin of centrality. Average critical m values are plotted only if the value is based on at least 10 nodes.

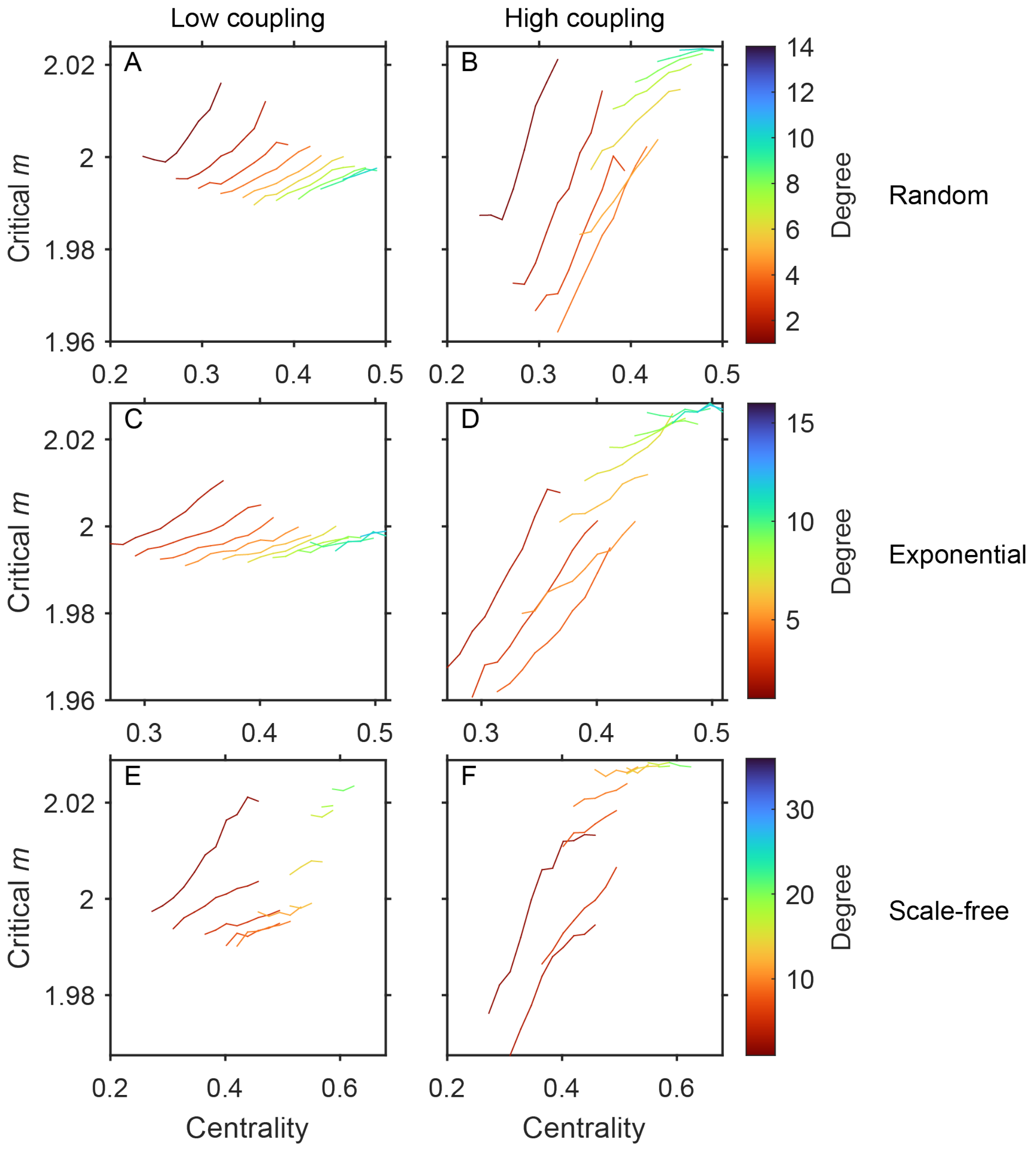

Figure S3. Results as in Figure S1, here represented as the effect of the centrality of the perturbed node on the vulnerability. Each curve represents the results for a given bin of degree. Average critical *m* values are plotted only if the value is based on at least 10 nodes.

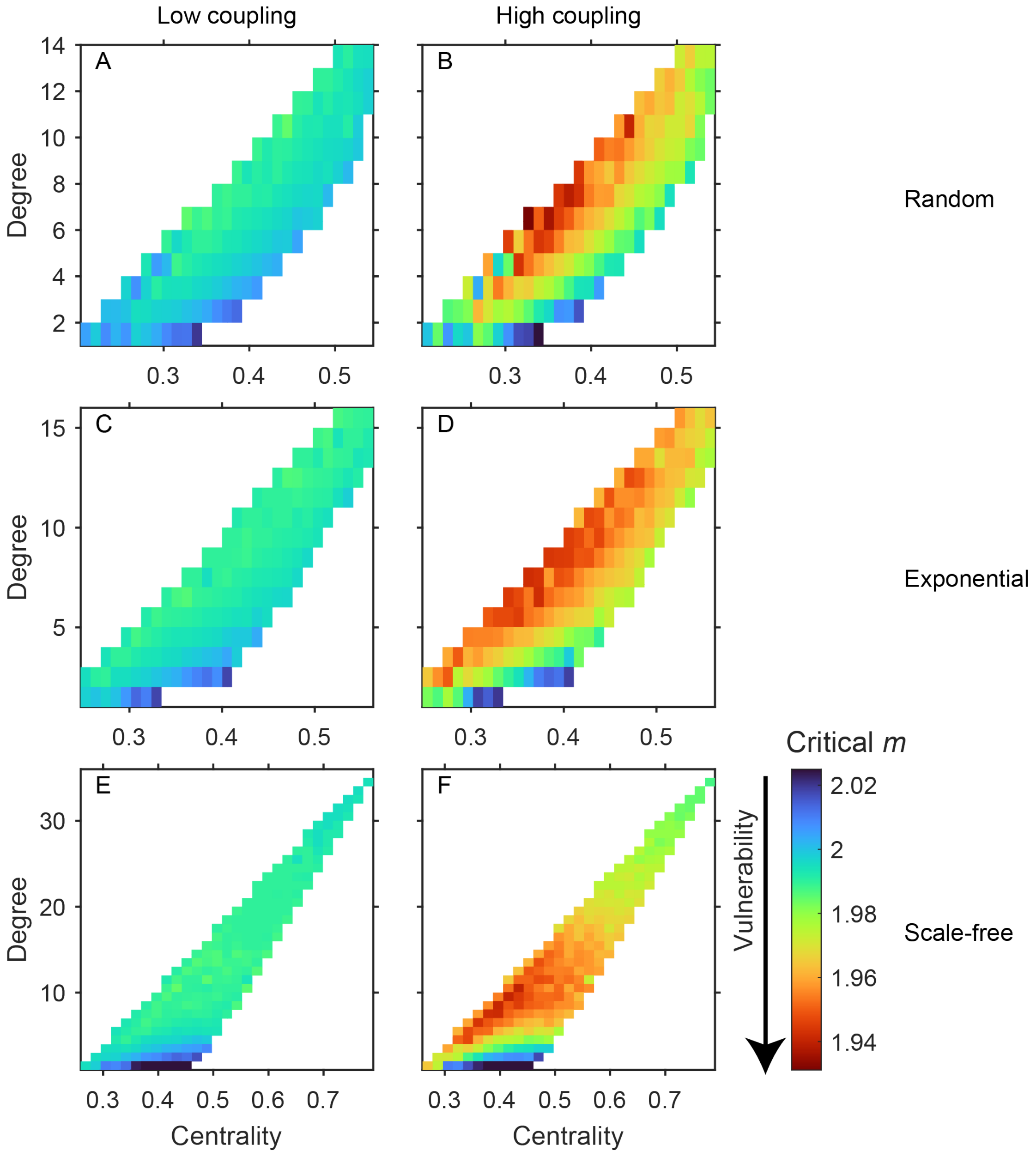

Figure S4. Effect of centrality and the number of connections ('degree') on the vulnerability of a node to trigger a systemic transition in the network of the deterministic Allee-effect model. In contrast to Figure S1, here, the studied nodes are continuously perturbed through setting the mortality rate high locally ($m_i = 4$). Different panels represent different network architectures. Red shading represents a high vulnerability in the sense that a systemic transition can be triggered from a node in this position even if the network is quite resilient (corresponding to a low value of parameter m_i). A. Random network with low coupling strength ($d = 0.05$); B. Random network with high coupling strength ($d = 0.15$). C. Exponential network with low coupling strength ($d = 0.05$); D. Exponential network with high coupling strength ($d = 0.15$). E. Scale-free network with low coupling strength ($d = 0.05$); F. Scale-free network with high coupling strength ($d = 0.15$); Other parameter values as in Table S1.

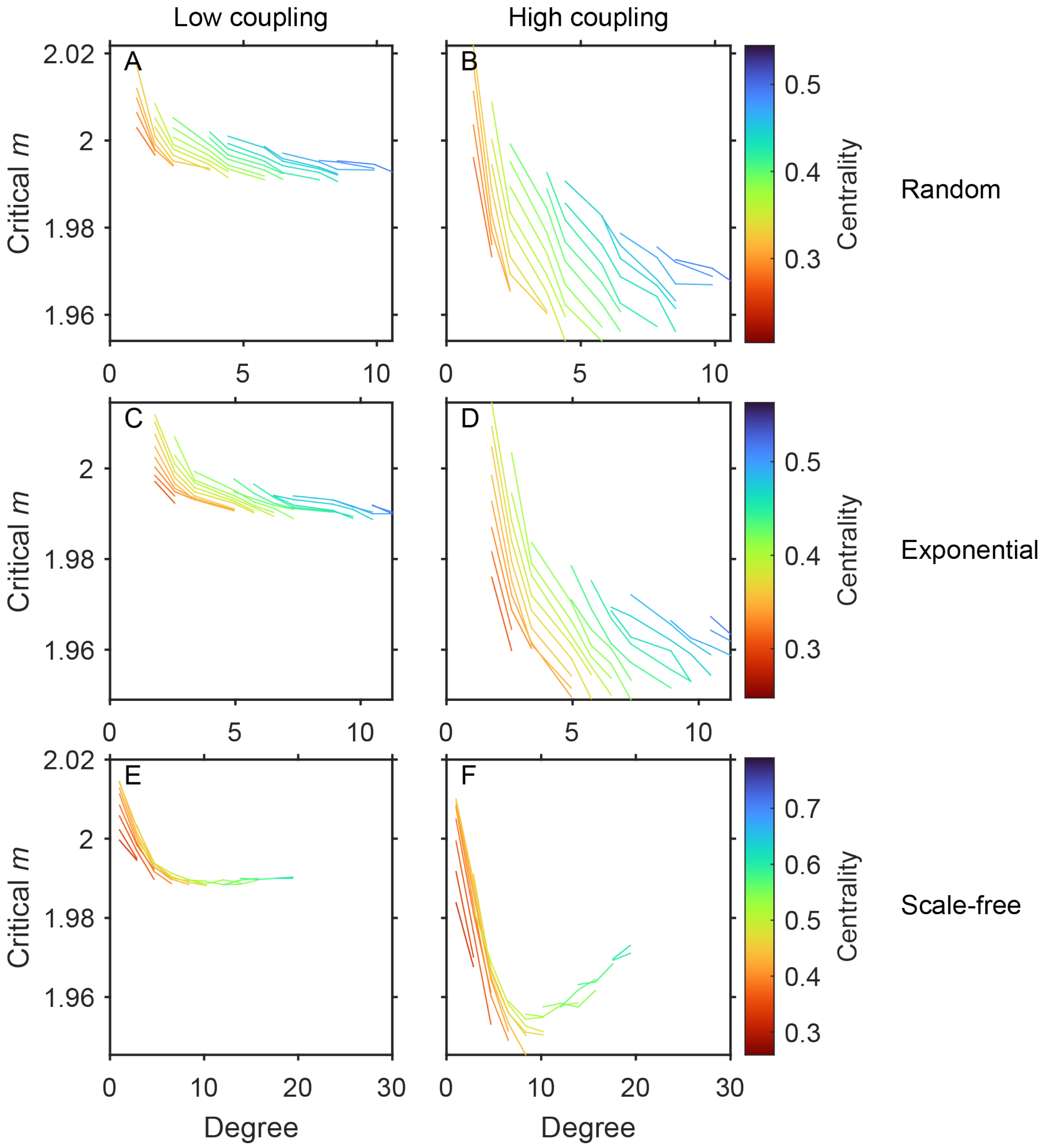

Figure S5. Results as in Figure S4, here represented as the effect of the degree of the perturbed node on the vulnerability. Each curve represents the results for a given bin of centrality. Note that panel F is the only one that still has a minimum. This may be understood from the fact that the central node in a scale free network has so many neighbors that it can hardly collapse. This effect is possibly due to the fact that we did not keep the state value fixed, but instead increased the mortality (in this case to a value of 4. With a higher mortality in the central node, this effect will be weaker or even disappear). Average critical *m* values are plotted only if the value is based on at least 10 nodes.

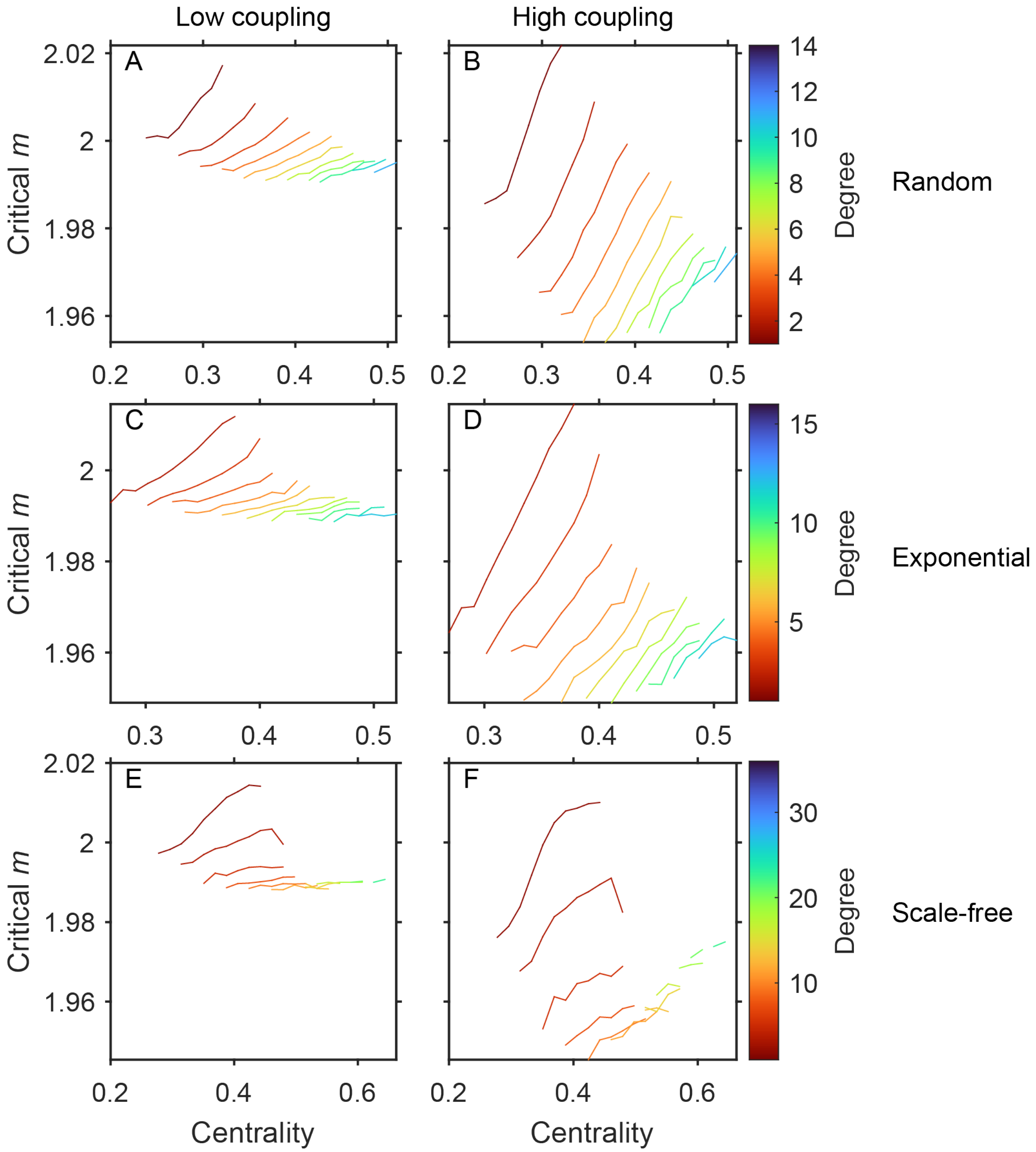

Figure S6. Results as in Figure S4, here represented as the effect of the centrality of the perturbed node on the vulnerability. Each curve represents the results for a given bin of degree. Average critical *m* values are plotted only if the value is based on at least 10 nodes.

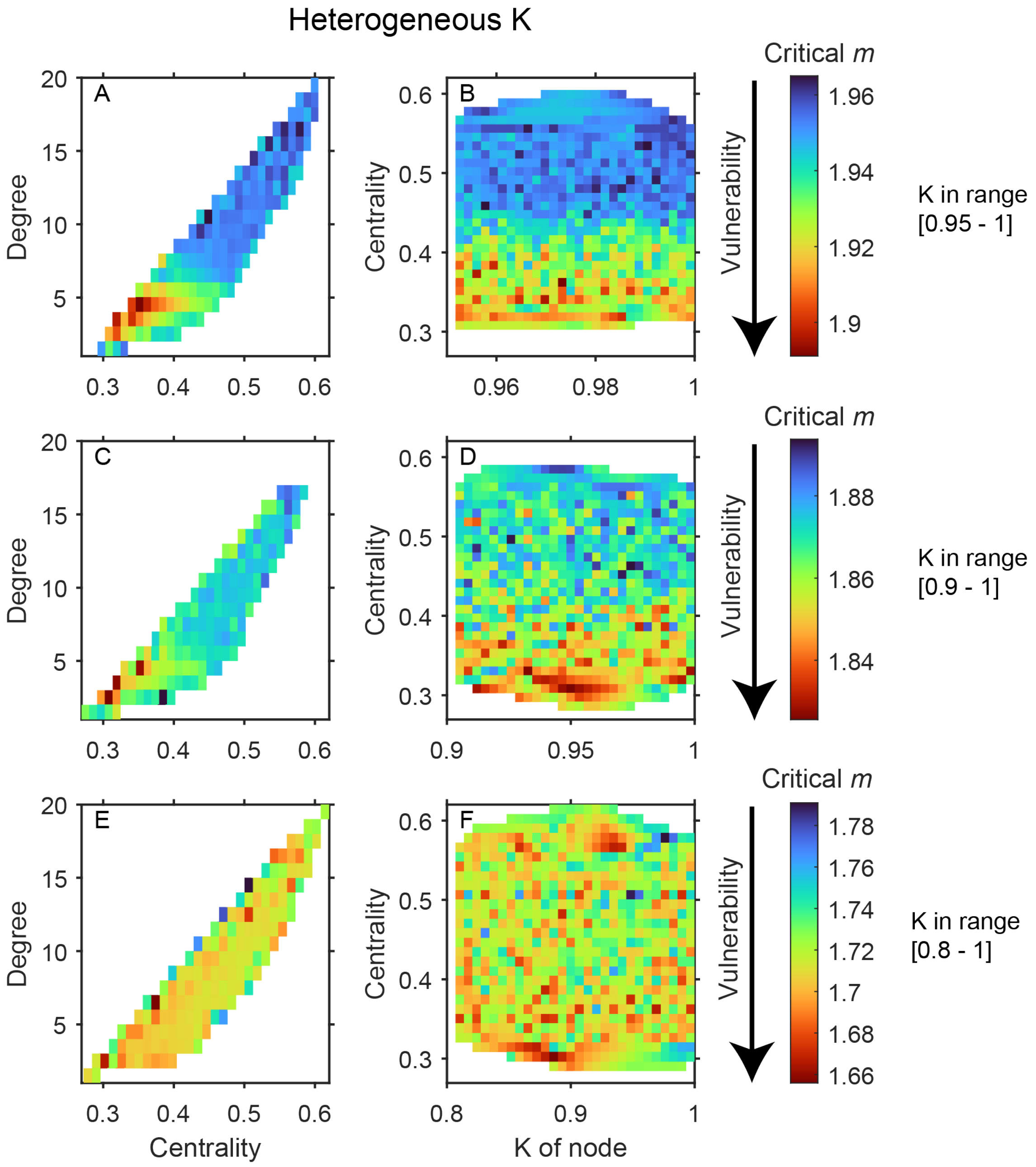

Figure S7. Effect of a heterogeneous network. Here, we simulated the exponential network with high coupling, randomly assigning a K drawn from a uniform distribution to each node (see the ranges on right side of the graph). Note that the results hold qualitatively if K is allowed to vary within 5 (A-B) and 10% (C-D). If the range of K is larger (e.g. 20% in E-F), the effect of the location of the initially perturbed node is overwhelmed by other local effects.

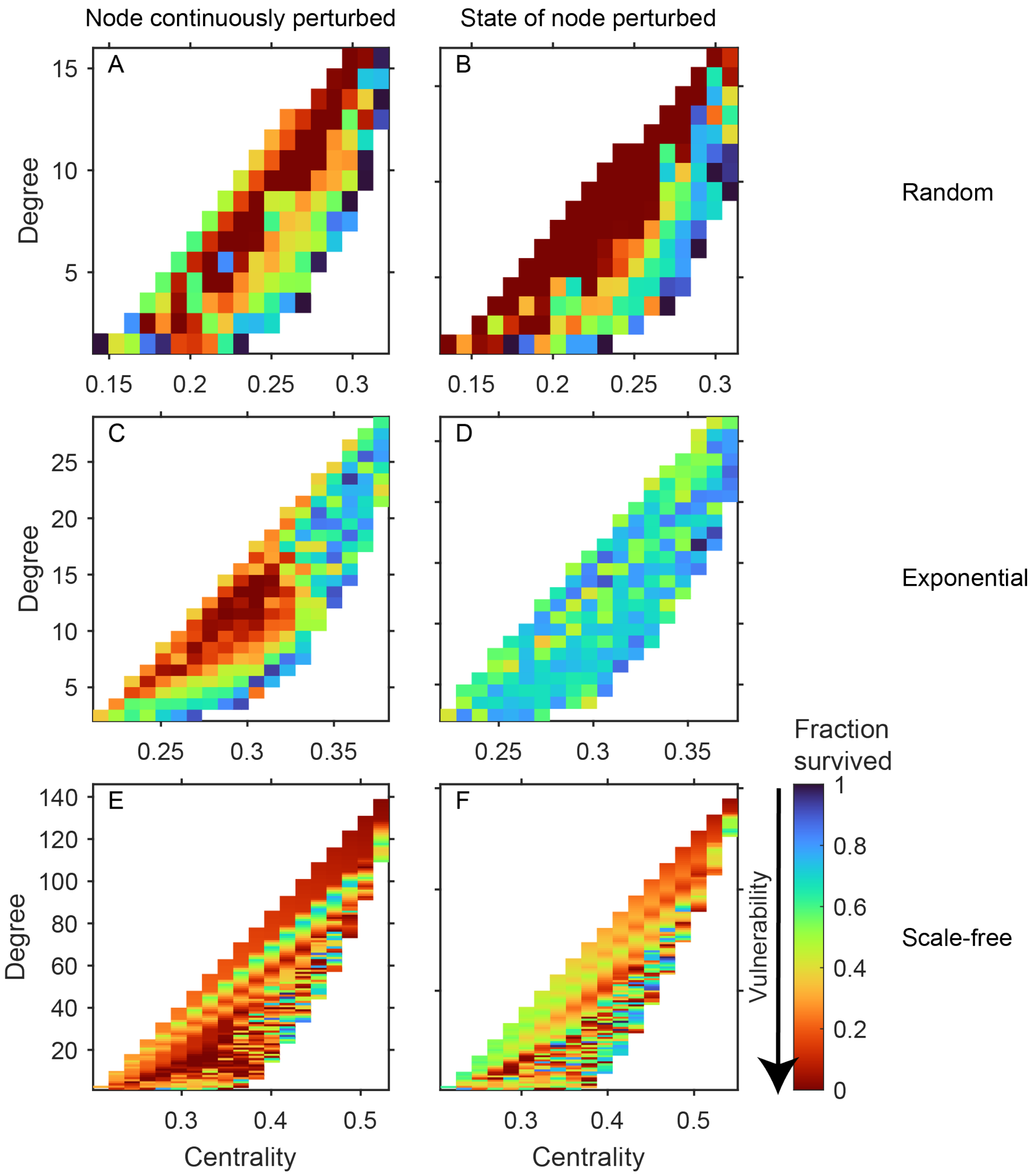

Figure S8. Vulnerability of a network to a local perturbation, depending on the degree and the centrality of a single perturbed node based on the stochastic Ising model. The colors represent the fraction of runs in which less than 50% of the nodes in the network has shifted. Different panels represent different network architectures. A-B. Random network (h = 1.3), C-D. Exponential network (h = 1.3), E-F. Scale-free network (h = 1.2). The nodes in this model were perturbated in two different ways. Left panels: nodes are flipped at start of the experiment, combined with press perturbation, locally h_i is set to 10; right panels: state of the node is flipped at start of experiment. Other parameter values as in Table S1.

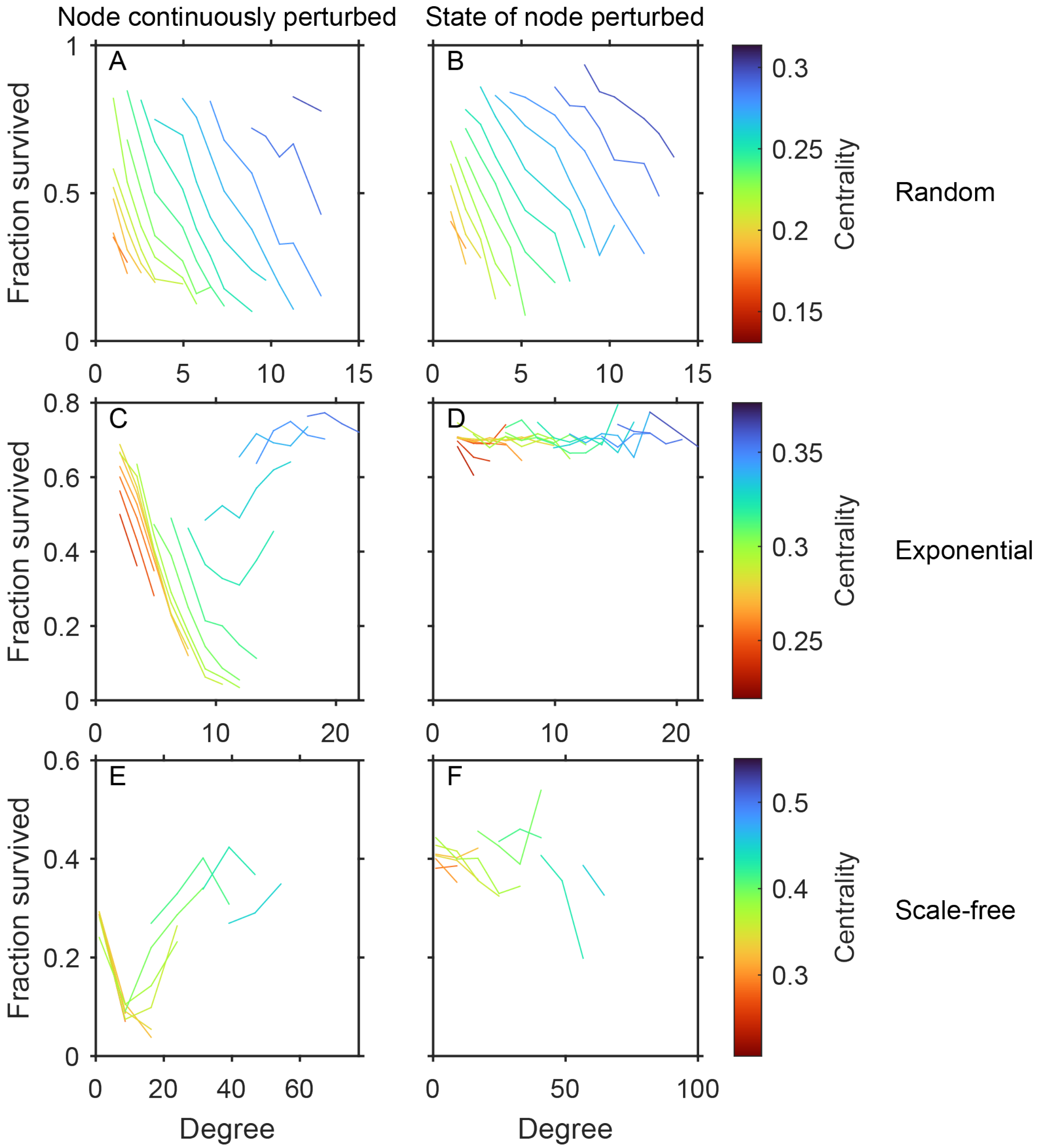

Figure S9. Results as in Figure S8, here represented as the effect of the degree of the perturbed node on the vulnerability, in terms of the fraction of runs in which less than 50% of the nodes in the network has shifted. The average fraction survived is plotted only if the value is based on at least 10 nodes.

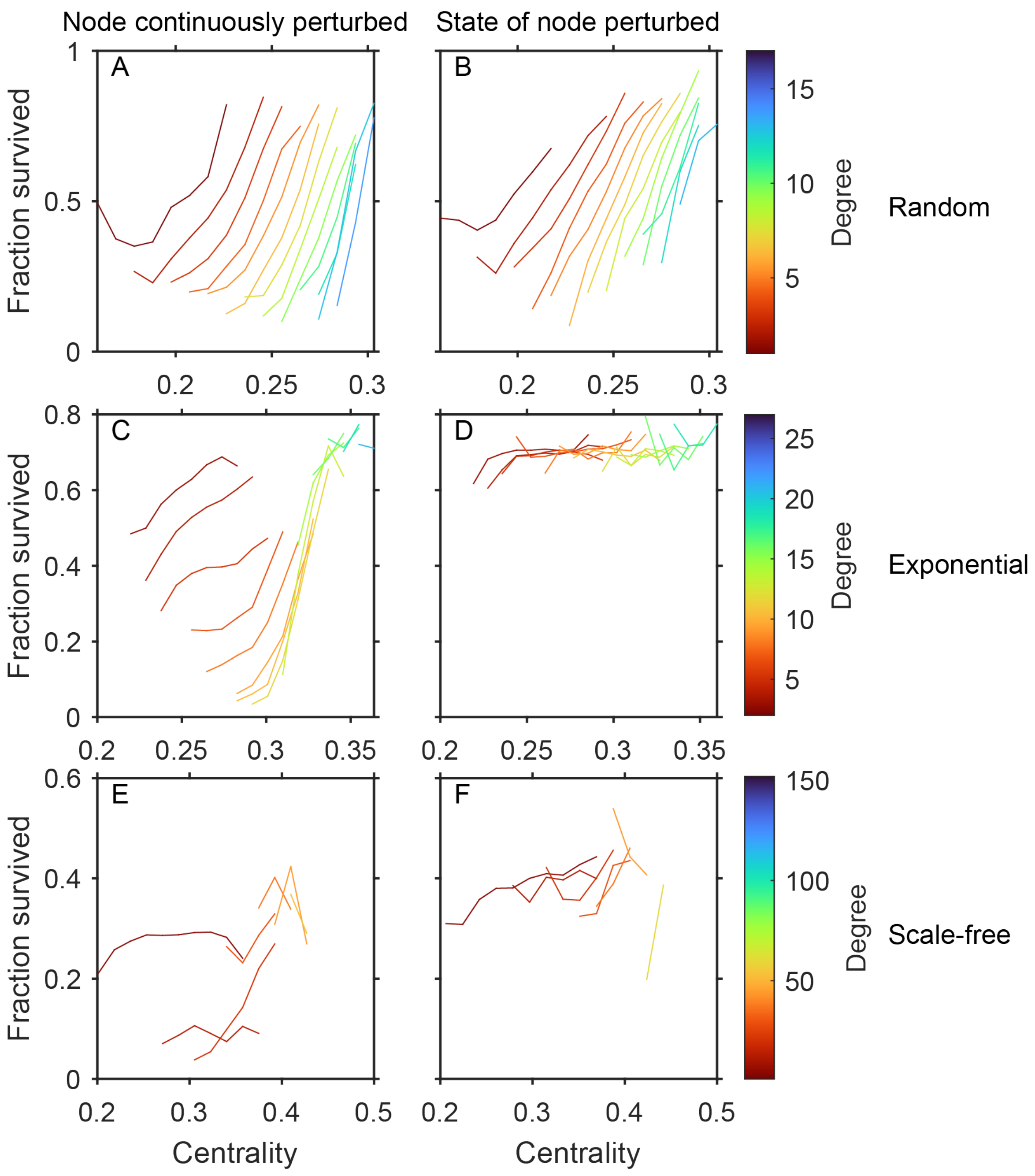

Figure S10. Results as in Figure S8, here represented as the effect of the centrality of the perturbed node on the vulnerability, in terms of the fraction of runs in which less than 50% of the nodes in the network has shifted. The average fraction survived is plotted only if the value is based on at least 10 nodes.

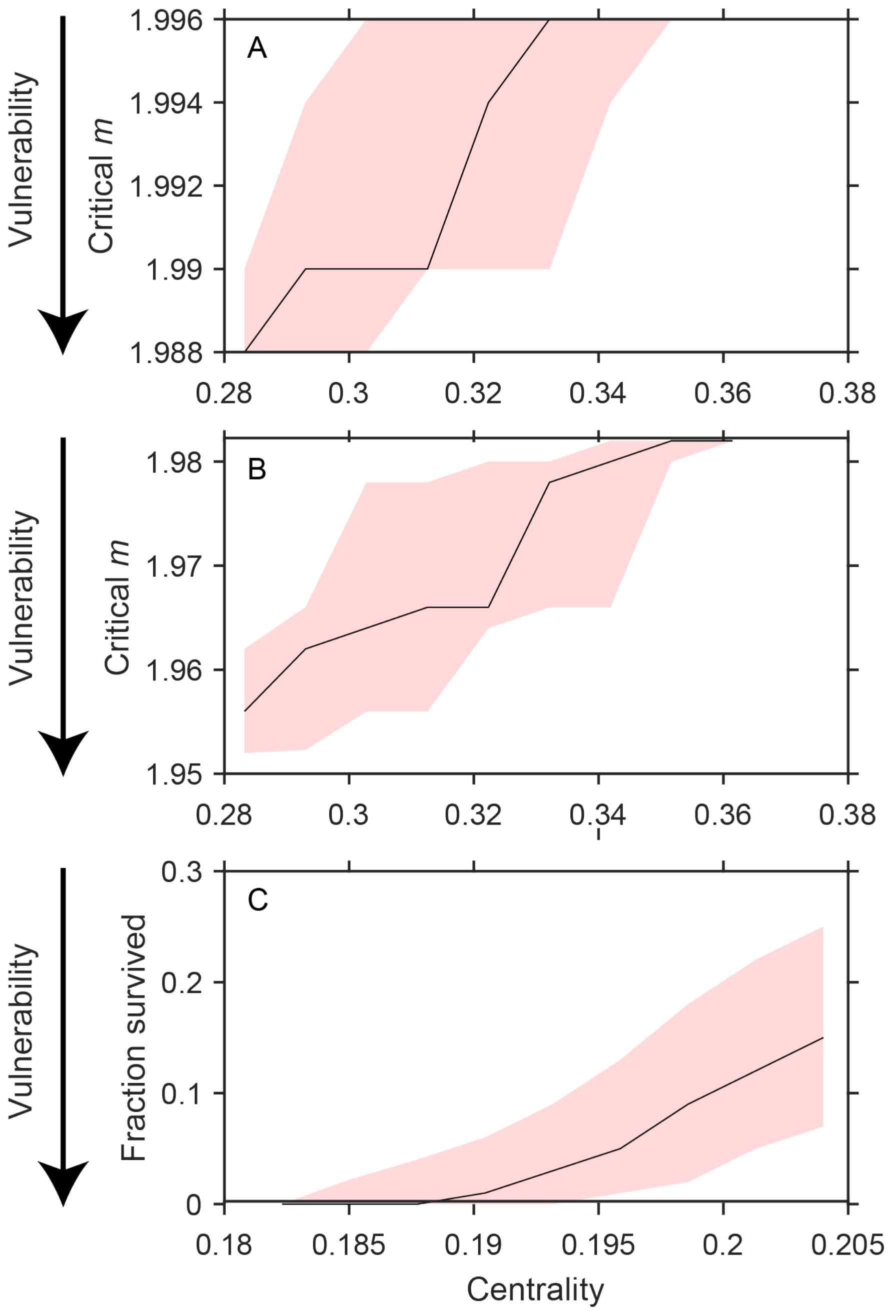

Figure S11. Vulnerability of random-regular networks to a local perturbation, depending on the centrality of a single perturbed node. In these networks, the degree for all nodes is equal to 4. The black lines are the median of classes of centrality, the shaded areas show the range between the 5% and 95% percentiles. A. Deterministic Allee-effect model, low coupling strength (d=0.05), state of node perturbed. B. Deterministic Allee-effect model, high coupling strength (d=0.15), state of node perturbed. C. Stochastic Ising model, nodes continuously perturbed.

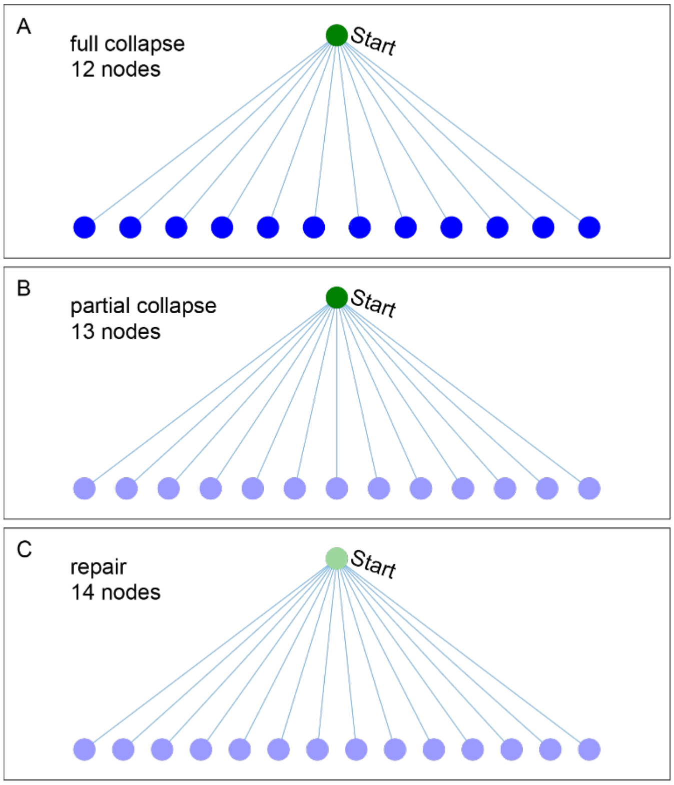

Figure S12. Local critical mass barriers to spread of a perturbation in simple networks (deterministic Allee effect model). At the start of the simulation all nodes are in the high biomass equilibrium except for the green start node, which initially has no biomass. In panel A, all 12 nodes collapse, in panel B, only the start node remains in the low biomass state, and all other 13 nodes are still in the high biomass state. In panel C the start node is repaired by the 14 neighboring nodes, so all nodes are at the end of the simulation in the high biomass state. Parameter values: m=1.99; d=0.05 (others as in Table S1).

References

46 Ising, E. Beitrag zur Theorie des Ferromagnetismus. *Zeitschrift für Physik* **31**, 253-258 (1925). https://doi.org:10.1007/bf02980577

47 van Borkulo, C. *et al.* Association of symptom network structure with the course of depression. *JAMA psychiatry* **72** (2015).

48 Brock, W. & Durlauf, S. A formal model of theory choice in science. *Economic Theory* **14**, 113-130 (1999).

49 Scheffer, M., Westley, F. & Brock, W. Slow response of societies to new problems: Causes and costs. *Ecosystems* **6**, 493-502 (2003). https://doi.org:DOI 10.1007/s10021-002-0146-0

50 Kotze, J. Introduction to Monte Carlo methods for an Ising Model of a Ferromagnet. *arXiv preprint arXiv:0803.0217* (2008).

51 Barabási, A.-L. & Pósfai, M. *Network science*. (Cambridge University Press, 2016).

52 Albert, R. & Barabási, A.-L. Statistical mechanics of complex networks. *Rev. Mod. Phys.* **74**, 47-97 (2002). https://doi.org:10.1103/RevModPhys.74.47

53 Barabasi, A. L. & Albert, R. Emergence of scaling in random networks. *Science* **286**, 509-512 (1999). https://doi.org:10.1126/science.286.5439.509

54 Bollobás, B. *Random Graphs*. (Cambridge University Press, 2001).
